# Morphological and cytogenetic characteristics of Neobisium (Blothrus) slovacum Gulička, 1977 (Pseudoscorpiones, Neobisiidae), the northernmost troglobitic species of the subgenus Blothrus in Europe

**DOI:** 10.3897/zookeys.817.27189

**Published:** 2019-01-15

**Authors:** Martina Červená, František Šťáhlavský, Vladimír Papáč, Ľubomír Kováč, Christophoryová Jana

**Affiliations:** 1 Department of Zoology, Faculty of Natural Sciences, Comenius University, Mlynská dolina, Ilkovičova 6, SK-842 15 Bratislava, Slovakia Comenius University Bratislava Slovakia; 2 Department of Zoology, Faculty of Science, Charles University in Prague, Viničná 7, CZ-128 44, Prague 2, Czech Republic Charles University Prague Czech Republic; 3 Slovak Caves Administration, Železničná 31, SK-979 01 Rimavská Sobota, Slovakia Slovak Caves Administration Rimavská Sobota Slovakia; 4 Department of Zoology, Institute of Biology and Ecology, Faculty of Science, P.J. Šafárik University, Šrobárova 2, SK-041 54 Košice, Slovakia Šafárik University Košice Slovakia

**Keywords:** Distribution, endemic, FISH, sex chromosome, Slovak Karst, troglobitic, 18S rDNA

## Abstract

A redescription is provided of the adult, tritonymph and deutonymph life stages of the troglobitic Neobisium (Blothrus) slovacum Gulička, 1977, which is known from Slovakia and Hungary. Material examined included 35 previously deposited museum specimens and 15 newly collected specimens. In addition, the karyotype and distribution of 18S rDNA clusters are described, using fluorescence in situ hybridization (FISH). The male karyotype of *N.slovacum* comprises 69 chromosomes, with a predominance of biarmed chromosomes, and an X0 sex chromosome system. Two pairs of signals for 18S rDNA on biarmed chromosomes (submetacentric and metacentric) of different sizes were identified. The present study provides the first information about the distribution of these clusters in the arachnid order Pseudoscorpiones. The geographic distribution of the species is summarized and mapped. *Neobisiumslovacum* is endemic to the Slovak and Aggtelek Karst area in southern Slovakia and north-western Hungary, where it has been recorded from 16 caves. One of these, Hačavská cave (in Slovakia), is the northernmost locality known for any species of the subgenus Blothrus.

## Introduction

**Figure 1.**
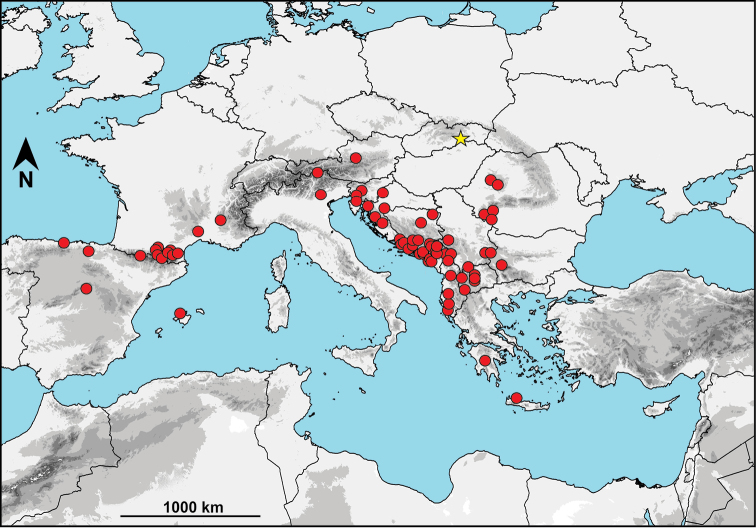
Distribution of the subgenus Blothrus in Europe. Red circles represent type localities of species. Yellow star indicates the northernmost occurrence of the subgenus in Slovakia (*Neobisiumslovacum*).

In Europe, troglobitic pseudoscorpions of the genus *Neobisium* Chamberlin, 1930 occur in four subgenera: *Blothrus* Schiödte, 1847, *Ommatoblothrus* Beier, 1956, *Heoblothrus* Beier, 1963 and *Pennobisium* Ćurčić, 1988. Of these, *Blothrus* is the most frequent genus in European caves (Ducháč and Mlejnek 2000a), and includes about 90 species (Harvey 2013). The majority of these species are distributed in southern Europe (Harvey 2013; Fig. 1), with only six species occurring in the Carpathian Mountains (Novák 2014).

In Slovakia, the sole species of the subgenus Blothrus is *Neobisiumslovacum* Gulička, 1977, which has clear troglomorphic characters (Gulička 1977a, b). This species is endemic to the Western Carpathians (Gulička 1977a, Ducháč and Mlejnek 2000a), with a distribution limited to the Slovak and Aggtelek Karst, a well-known karst area on the border between Slovakia and Hungary. This species was first collected by Lysenko in 1966 in the Diviačia chasm (Lysenko 1972), but it was not identified at that time. The specimens were rediscovered in Dr P.H. Verner’s collection thirty years later and correctly assigned to this species by Ducháč (Ducháč and Mlejnek 2000a, b).

*Neobisiumslovacum* is a strictly troglobitic species, with specific morphological adaptations to the cave environment. Compared to epigean relatives, the body of *N.slovacum* is pale, the legs and pedipalps are elongated, and eyes are absent. The remarkably elongated appendages provide an advantage in locating and capturing prey in the cave environment. The specimens are usually found on walls, in stony debris with clay sediments and near organic material (bat guano) in caves (Kováč et al. 2010).

The species was originally described from Stará brzotínska cave by Gulička (1977a), based on an adult holotype (sex not indicated) and two paratype nymphs. The description of the type material was incomplete because it omitted many important features necessary for proper species delimitation and identification. The depository of the type material is not known. Ducháč (1996) examined an additional specimen from the type locality and added some characteristics that were not reported in the original description. However, some morphological features useful for identification remained unknown. Ducháč (2002) summarized all previous collections of *N.slovacum* in his Ph.D. thesis, which contained descriptions of 53 adults, two tritonymphs and one deutonymph. However, these were still incomplete, with characters such as the chaetotaxy of sternites, morphometric data of legs I and IV, and the number of teeth on the cheliceral and palpal fingers not being specified.

Later, Ducháč (2004a, b) described in detail the male genitalia and the chaetotaxy of the genital opercula of *N.slovacum* and provided partial morphological descriptions of deutonymphs and tritonymphs, but the drawings and measurements were inadvertently omitted by the journal. Intraspecific variability of *N.slovacum* from the Slovak Karst was discussed by Ducháč (1999), who compared it with all Carpathian species of the subgenus Blothrus.

**Figure 2.**
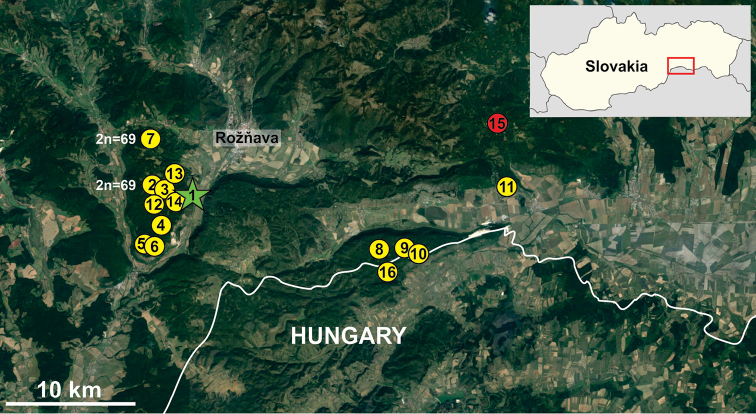
Distribution of *Neobisiumslovacum* in karst in Slovakia and Hungary. Green star indicates the type locality and red spot the northernmost locality of the subgenus Blothrus. The karyotype is recorded for two localities. Localities numbered as in Table 1.

After Stará brzotínska cave, the species was found at 14 other localities in the Slovak Karst (Fig. 2, Table 1). Of these, Šingliarova chasm was the northernmost locality for the species, as well as for subgenus. Outside Slovakia, *N.slovacum* has been recorded from Meteor cave in the Aggtelek Karst in Hungary (Ducháč and Mlejnek 2000b). The distribution of *N.slovacum* in caves of the Slovak and Aggtelek Karst was discussed by Ducháč and Mlejnek (2000a, b). More recent findings of *N.slovacum* have been published by Vlk (2001) from Natrhnutá chasm, by Christophoryová (2009) from Šingliarova chasm, and by Kováč et al. (2010) from Slnečná, Vlčia and Veľká Peňažnica chasms.

**Table 1.** List of collection localities of *Neobisiumslovacum* in the Slovak Karst area (1–15 Slovakia; 16 Hungary). Abbreviations: a.s.l., above sea level; N, latitude; W, longitude (see map, Fig. 2).

Code	Locality	N	W	m a.s.l.
1	Stará brzotínska cave (type locality)	48°36'32", 20°28'15"		258
2	Zvonivá chasm	48°37'04", 20°25'33"		675
3	Zombor chasm	48°36'48", 20°26'38"		645
4	Diviačia chasm	48°35'02", 20°26'30"		597
5	Čikova diera cave	48°34'14", 20°24'50"		526
6	Fialová cave	48°34'12", 20°24'55"		544
7	Šingliarova chasm	48°39'21", 20°25'04"		677
8	Obrovská chasm	48°34'04", 20°41'05"		535
9	Pri salaši 2 chasm	48°34'21", 20°43'20"		543
10	Natrhnutá chasm	48°34'00", 20°43'56"		508
11	Erňa cave	48°36'56", 20°50'36"		410
12	Slnečná chasm	48°35'60", 20°24'83"		564
13	Vlčia chasm	48°36'89", 20°27'05"		660
14	Veľká Peňažnica chasm	48°35'83", 20°27'12"		667
15	Hačavská cave	48°39'50", 20°49'47"		795
16	Meteor cave	48°33'11", 20°42'26"		443

Although *N.slovacum* seems to be restricted to the cave systems of the Slovak and Aggtelek Karst area, the possibility exists that the isolation of populations in separate cave systems could have led to diversification and cryptic speciation in this region. The detection of cryptic species diversity has recently been based mainly on the analysis of mitochondrial and nuclear gene sequences in terrestrial (e.g. Opatova and Arnedo 2014) and subterranean (e.g. Harrison et al. 2014) arachnid groups. However, cytogenetic techniques can also be useful for the identification of cryptic species within various orders of arachnids, such as harvestmen (Šťáhlavský et al. 2018a), spiders (Řezáč et al. 2018), scorpions (Šťáhlavský et al. 2018b) and pseudoscorpions (Zaragoza and Šťáhlavský 2008). This is especially notable in some species of edaphic pseudoscorpions characterized by limited dispersal ability, for which distinct karyotypes may be found even in very close localities (Kotrbová et al. 2016). It is therefore useful to combine descriptions the external morphology with karyotype analysis (Plíšková et al. 2016), as is done here.

Summarizing the previously published data, 61 adults and six nymphs have been collected from Slovakia. Despite this, knowledge of the morphology of *N.slovacum* remained incomplete. All previous descriptions were deficient in important details. Consequently, the aims of the present study are to: (1) complete and examine previously studied material of *N.slovacum*, (2) describe newly collected specimens and provide additional information on the variability of morphometric and morphological characters, (3) analyse the *N.slovacum* karyotype and (4) analyse the distribution of *N.slovacum* in caves of the Western Carpathians.

## Methods

To locate previously studied material of *N.slovacum*, the natural history museums in Vienna, Bratislava and Berlin, and the zoology departments of Charles University in Prague and Comenius University in Bratislava were contacted. In total, 35 specimens from eight localities in the Slovak Karst were obtained, including one specimen from the type locality. These specimens have been deposited in the Department of Zoology of Charles University in Prague and the Natural History Museum in Vienna (NHMW). The recently collected specimens of *N.slovacum* (15 specimens) were obtained at three localities of the Slovak Karst: Šingliarova chasm, Hačavská cave and Zvonivá chasm. The new material from Hačavská cave and Šingliarova chasm was deposited in the Natural History Museum in Vienna (NHMW) and material from the Zvonivá chasm in the Department of Zoology, Charles University in Prague.

Recently collected specimens were identified using the identification keys in Beier (1963), Christophoryová et al. (2011) and Novák (2014). Morphological terminology follows Chamberlin (1931) and Judson (2007); nomenclature follows Harvey (2013). All specimens were studied as temporary slide mounts using lactic acid as the medium, then rinsed in water and returned to 70% ethanol. Morphological and morphometric analyses were performed using a Leica DM1000 compound microscope with ICC50 Camera Module (LAS EZ application, 1.8.0). Measurements (in mm) were taken from digital images using the AxioVision 40LE application. Reference points for measurements follow Chamberlin (1931).

### Cytogenetic analysis

One male from Zvonivá chasm (locality 2: Table 1 and Fig. 2) and two males from Šingliarova chasm (locality 7: Table 1 and Fig. 2) were used for the cytogenetic analysis.

The chromosomes were prepared by the “spreading” method described in Šťáhlavský and Král (2004), slightly modified as noted below. The dissected gonads were hypotonized in 0.075 M KCl for 30 min and then fixed in a methanol:glacial acetic acid (3:1) solution for at least 20 min. The tissue was then dissociated in a drop of 60% acetic acid on a clean microscope slide and the suspension was moved with tungsten needles on the surface until the fluid evaporated. The chromosomes were stained with 5% Giemsa solution in Sörensen phosphate buffer for 30 min. Chromosomes were documented using an Olympus IX81 inverted microscope equipped with a Hamamatsu ORCA-AG monochromatic camera. In total, ten metaphases II were measured using the LEVAN plugin (Sakamoto and Zacaro 2009) for IMAGEJ 1.47 software (http://imagej.nih.gov/ij/). For the identification of rDNA clusters, FISH with an 18S rDNA probe was used for all three males. This probe was prepared from the scorpion *Euscorpiussicanus* (Koch, 1837), as described by Šťáhlavský et al. (2018a). The probe was labelled by PCR with biotin-14-dUTP (Roche) using a Nick Translation Kit (Abbott Molecular) following the manufacturer’s guidelines. The FISH protocol was performed following Forman et al. (2013), on the same chromosome preparation used for the standard karyotype analysis after washing Giemsa in methanol:glacial acetic acid (3:1) solution. During the FISH procedure, the slides were treated with RNase A (200 µg/ml in 2× saline-sodium citrate) (60 min, 37 °C). The chromosomes were then denatured in 70% formamide in 2× saline-sodium citrate (3 min 30 s, 68 °C). Finally, the probe mixture (20 ng of probe, 25 ng of salmon sperm DNA (Sigma Aldrich), 10 µl of 50% formamide, 10% dextran sulphate in 2× saline-sodium citrate) was applied to each slide and hybridization was performed overnight in a black box at 37 °C. The following day, the probe was detected with Cy3-conjugated streptavidin. The signal was intensified by application of biotinylated anti-streptavidin and Cy3-conjugated streptavidin. Chromosomes were counterstained with DAPI (Fluoroshield, Sigma-Aldrich) and documented using an Olympus IX81 microscope equipped with a Hamamatsu ORCA-AG monochromatic CCD camera. The images were pseudocoloured (red for Cy3 and blue for DAPI) and superimposed with Cell^R software (Olympus Soft Imaging Solutions GmbH).

## Results

### Neobisium (Blothrus) slovacum

Taxon classificationAnimaliaPseudoscorpionesNeobisiidae

Gulička, 1977

Figures 3 , 4 , 5

Neobisium (Blothrus) slovacum Gulička, 1977a: 6–8, figs 1–4; Gulička 1977b: 24, fig. 1; Harvey 1991: 383; Ducháč 1996: 154–157, figs 1–9; Ducháč 1999: 176–179, figs 1–2, 6–8; Ducháč and Mlejnek 2000a: 48–49, figs 1–2; Ducháč and Mlejnek 2000b: 19–20, figs 1–2; Ducháč 2004a: 53–54, figs 1–2; Ducháč 2004b: figs 1–10, table 1; Kárpáthegyi 2007: 86; Christophoryová et al. 2011: 36, fig. 3D; Novák 2014: 394–400.

#### Diagnosis (adults).

*Neobisiumslovacum* is an eyeless, troglobitic species that differs from other Carpathian species of the subgenus Blothrus in following combination of characters: posterior margin of carapace usually with 4 setae; a subocular seta usually present (sometimes missing on one side); epistome absent; cheliceral hand with 5 setae; anterolateral process of coxa I long, broad and apically pointed, medial process prominent, rounded, with strong denticles; palpal trochanter without tubercles; palpal femur ratio in the range 5.69–8.81 mm; fixed palpal finger with unequally long teeth; telotarsus IV with 2 long tactile setae. Chromosomes of male 2n = 69.

**Figure 3.**
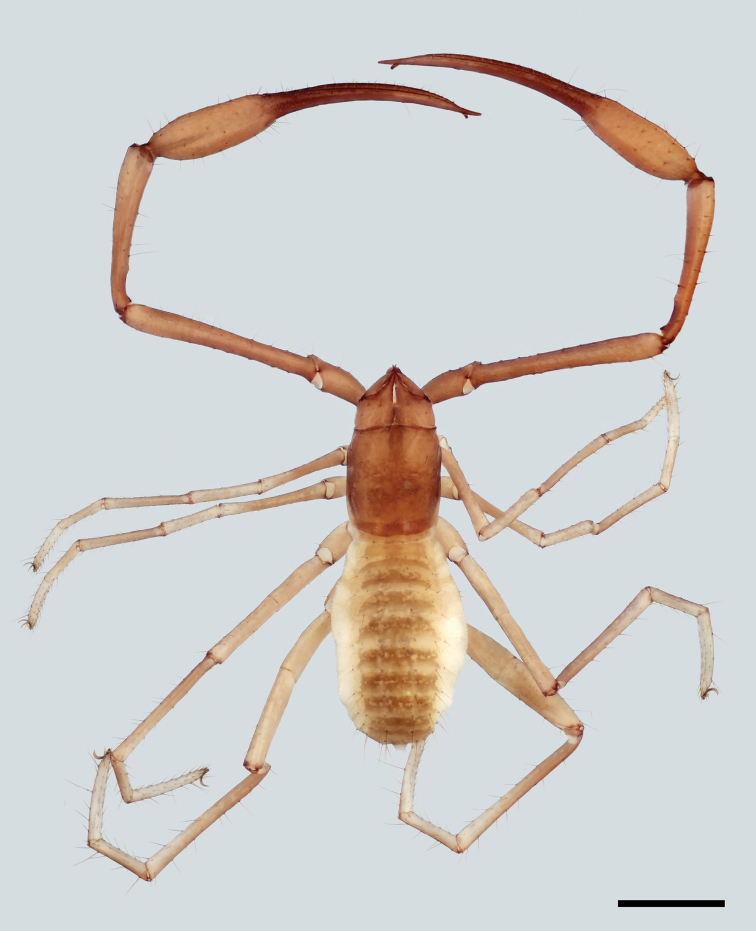
Male of *Neobisiumslovacum* from Hačavská cave. Scale line: 1 mm.

#### Type locality.

Slovakia, Slovak Karst, Stará brzotínska cave (48°36'32"N; 20°28'15"E, 258 m a.s.l., length 120 m).

#### Holotype.

Adult of undetermined sex collected from stony debris on clay sediment on 26 September 1974. Apparently lost.

#### Paratypes.

2 nymphs, from type locality, collected on 10 August 1975. Apparently lost.

#### Note.

The depository of the type material is unknown. Searches for the holotype and paratypes in various institutions (listed in Methods section) were unsuccessful.

#### Material examined

(see Table 1 for coordinates and altitudes of localities):

#### Previously studied material.

**Čikova diera cave** (length 60 m, depth 26 m): 1 ♀ (det. V. Ducháč), 21 February–13 March 1988, leg. R. Mlejnek; 1 ♂ (det. V. Ducháč), 12 May 1988, leg. R. Mlejnek; 1 ♀ (det. V. Ducháč), 9 June 1989, leg. R. Mlejnek; **Diviačia chasm** (length 468 m, depth 127 m): 1 ♂, 2 ♀ (det. V. Ducháč), 1966, leg. V. Lysenko; **Erňa cave** (length 60 m, depth 10 m): 2 ♀, 1 specimen represented by appendages only, sex unknown (det. V. Ducháč), 20 July 1999, leg. R. Mlejnek; **Fialová cave** (length 21 m, depth 5.5 m): 1 ♀ (det. V. Ducháč), 15 April 1989, leg. R. Mlejnek; 1 ♂ (det. V. Ducháč), 23 September 1989, leg. R. Mlejnek; 1 ♂, 1 ♀, 1 tritonymph (det. V. Ducháč), 17 January 1998, leg. R. Mlejnek; 1 ♂ (det. V. Ducháč), 11 May 1998, leg. R. Mlejnek; **Obrovská chasm** (depth 100 m): 1 ♂ (det. V. Ducháč), 8 May 1988, leg. R. Mlejnek; **Pri salaši 2 chasm** (length 50 m, depth 36 m): 1 specimen represented by appendages only (det. V. Ducháč), 8 May 1988, leg. R. Mlejnek; **Stará brzotínska cave** (length 120 m): 1 ♀ (NHMW 28661) (det. V. Ducháč), 6 June 1982, leg. P. Moravec; **Šingliarova chasm** (length 140 m, depth 72 m): 3 ♂, 1 ♀ and 1 ♀ damaged badly (det. V. Ducháč), 12 May 1988, leg. R. Mlejnek; 1 ♂ (det. V. Ducháč), 7 June–19 October 1998, leg. R. Mlejnek; 3 ♂, 3 ♀, 2 specimens (sex unknown) represented by appendages only (det. V. Ducháč), 16 May 1998, leg. R. Mlejnek; **Unknown locality**: 1 ♂ (NHMW 28664), 2 ♀ (NHMW 28664) (det. V. Ducháč), 4 March 2003, leg. R. Mlejnek, locality data missing.

#### Remarks.

Faunistic data and descriptions of the 35 specimens listed above were provided by Ducháč (1996, 1999, 2002, 2004a, b) and Ducháč and Mlejnek (2000a, b). It was generally not possible to correlate specimens with an individual literature source, because only the numbers of individuals and variability of some characters were mentioned. Of these specimens, 27 were used in the present study to add information on previously ignored characters. The remaining eight specimens were not examined in detail, either because the locality was unknown or because they were not in a suitable condition.

#### Newly obtained material.

**Hačavská cave** (length 200 m): 2 ♂, 1 ♀ (NHMW 28662); hand sampling, in stony debris with clay sediment, back part of the cave, 150 m in the cave from entrance, 3 April 2017, leg. V. Papáč; **Šingliarova chasm** (length 140 m, depth 72 m): 1 ♂, 2 ♀, 1 deutonymph (NHMW 28659), pitfall trapping, 11 October 2003, leg. A. Mock; 2 ♂, 1 ♀, hand sampling, 2 May 2007, leg. Ľ. Kováč; 2 ♂, 1 ♀ (NHMW 28660), hand sampling, on cave walls and rocks, Second Hall, 25 August 2012, leg. P. Ľuptáčik; **Zvonivá chasm** (length 494 m): 2 ♀, hand sampling, 5 May 2006, leg. Ľ. Kováč.

#### Cytogenetic analysis.

**Šingliarova chasm**: 2 May 2007: 2 ♂, hand sampling, leg. Ľ. Kováč; **Zvonivá chasm**: 5 May 2006: 1 ♂, hand sampling, leg. Ľ. Kováč.

#### Remarks.

**Figure 4.**
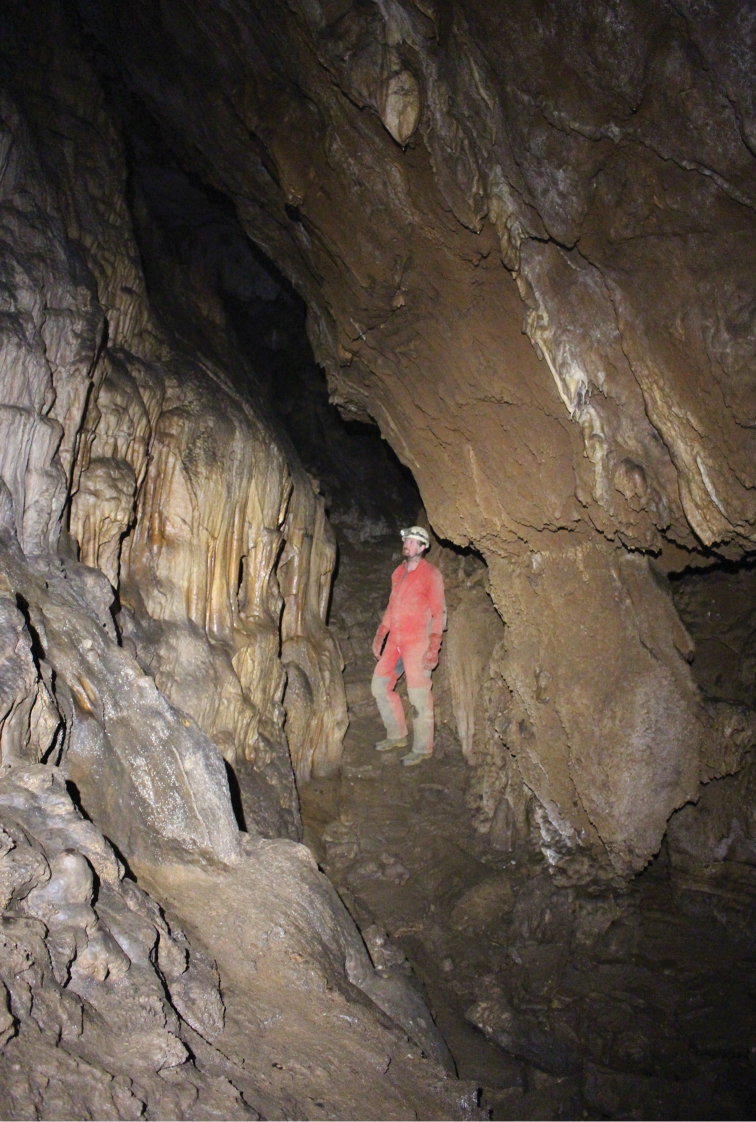
Back part of Hačavská cave, a new locality for the occurrence of *N.slovacum* in the Slovak Karst (photograph: V. Papáč); northernmost known locality of a member of the subgenus. Indicated as a yellow star on map (Fig. 1).

The newly collected specimens are described here, except for those used in the cytogenetic analyse. The record of the four specimens (NHMW 28659) from Šingliarova chasm was previously published by Christophoryová (2009). Hačavská cave represents a new locality for the species in Slovakia and is the northernmost locality of any member of the subgenus Blothrus (Figs 2, 4). In total, 14 adults and one deutonymph were identified in the new material from Šingliarova chasm, Hačavská cave and Zvonivá chasm.

#### Redescription.

In total, 40 adults, 1 tritonymph and 1 deutonymph were examined in the present study. Measurements of adults are given in Table 2.

**Table 2.** Morphometric data for males and females of *Neobisiumslovacum* (measurements in mm). Abbreviations: M, median; Min, minimum; Max, maximum; N, number of individuals measured; SD, standard deviation; x¯, arithmetic mean.

**Characteristics**	**Males**			**Females**		
	**Min–Max**	**M/x¯±SD**	**N**	**Min–Max**	**M/x¯±SD**	**N**
Body, length	2.80–3.97	3.54/3.18±1.05	11	3.00–3.90	3.38/3.42±0.30	14
Carapace, length	0.91–1.19	1.04/1.00±0.24	19	0.95–1.18	1.05/1.06±0.07	18
Carapace, posterior width	0.86–1.08	0.97/0.92±0.25	15	0.85–1.20	0.97/1.00±0.11	15
Carapace, length/posterior width ratio	0.98–1.13	1.07/1.01±0.28	15	0.92–1.24	1.08/1.08±0.09	14
Chelicera, length	0.61–0.78	0.69/0.66±0.16	19	0.67–0.80	0.69/0.71±0.04	18
Chelicera, width	0.29–0.40	0.36/0.34±0.08	20	0.35–0.43	0.37/0.38±0.02	19
Chelicera, length/width ratio	1.79–2.06	1.92/1.82±0.43	19	1.74–1.95	1.89/1.87±0.06	18
Cheliceral movable finger, length	0.37–0.49	0.44/0.42±0.10	20	0.42–0.50	0.46/0.46±0.02	19
Palpal trochanter, length	0.65–0.82	0.72/0.70±0.17	19	0.66–0.84	0.74/0.74±0.05	19
Palpal trochanter, width	0.20–0.35	0.28/0.26±0.07	20	0.25–0.35	0.29/0.29±0.03	20
Palpal trochanter, length/width ratio	2.03–3.09	2.58/2.50±0.65	19	2.09–2.89	2.50/2.54±0.18	19
Palpal femur, length	1.66–1.93	1.76/1.67±0.41	18	1.52–1.85	1.74/1.73±0.08	20
Palpal femur, width	0.20–0.27	0.22/0.21±0.05	20	0.19–0.32	0.23/0.23±0.03	20
Palpal femur, length/width ratio	6.52–8.81	7.96/7.38±1.87	18	5.69–8.74	7.65/7.60±0.74	20
Palpal patella, length	1.46–1.67	1.56/1.49±0.37	18	1.41–1.61	1.49/1.51±0.06	19
Palpal patella, width	0.20–0.30	0.24/0.24±0.06	20	0.22–0.32	0.26/0.26±0.03	20
Palpal patella, length/width ratio	5.20–6.75	6.17/5.79±1.48	18	5.00–6.83	5.88/5.82±0.53	19
Palpal hand, length with pedicel	1.01–1.32	1.17/1.12±0.27	19	1.05–1.25	1.15/1.16±0.05	19
Palpal hand, length without pedicel	1.01–1.14	1.06/1.01±0.26	17	0.90–1.14	1.04/1.04±0.06	19
Palpal hand, width	0.33–0.47	0.40/0.38±0.09	20	0.36–0.52	0.41/0.42±0.05	19
Palpal hand, length with pedicel/width ratio	2.43–3.40	2.95/2.82±0.70	19	2.21–3.19	2.80/2.76±0.26	18
Palpal finger, length	1.81–2.09	1.92/1.82±0.45	18	1.81–2.06	1.91/1.92±0.08	18
Palpal finger, length/palpal hand length with pedicel	1.52–1.69	1.61/1.53±0.37	18	1.59–1.77	1.66/1.66±0.05	16
Palpal chela, length	2.57–3.19	2.91/2.77±0.67	19	2.73–3.21	2.91/2.93±0.14	17
Palpal chela, width	0.33–0.47	0.40/0.38±0.09	20	0.36–0.52	0.41/0.42±0.05	17
Palpal chela, length/width ratio	6.43–7.94	7.33/6.97±1.70	19	5.67–8.08	7.04/6.99±0.63	15
Leg I trochanter, length	0.30–0.38	0.34/0.32±0.08	18	0.28–0.37	0.33/0.33±0.03	18
Leg I trochanter, depth	0.18–0.23	0.20/0.19±0.05	18	0.17–0.24	0.21/0.21±0.02	18
Leg I trochanter, length/depth ratio	1.48–1.79	1.60/1.53±0.39	17	1.33–1.94	1.55/1.62±0.17	17
Leg I femur, length	0.88–1.02	0.94/0.89±0.23	17	0.75–1.03	0.90/0.91±0.08	17
Leg I femur, depth	0.09–0.14	0.11/0.11±0.03	18	0.10–0.15	0.11/0.12±0.02	17
Leg I femur, length/depth ratio	6.79–9.78	8.18/7.83±2.10	17	6.67–9.36	7.70/7.84±0.79	17
Leg I patella, length	0.50–0.71	0.63/0.60±0.15	19	0.56–0.69	0.62/0.62±0.04	18
Leg I patella, depth	0.10–0.15	0.12/0.11±0.03	19	0.11–0.16	0.12/0.12±0.01	16
Leg I patella, length/depth ratio	4.20–6.80	5.32/5.11±1.38	19	4.00–6.00	5.12/5.07±0.53	16
Leg I tibia, length	0.64–0.82	0.73/0.69±0.17	18	0.65–0.77	0.72/0.71±0.04	19
Leg I tibia, depth	0.08–0.10	0.09/0.09±0.02	18	0.08–0.11	0.09/0.09±0.01	19
Leg I tibia, length/depth ratio	7.33–9.63	7.95/7.67±1.99	17	6.50–9.38	7.67/7.90±0.84	19
Leg I basitarsus, length	0.40–0.49	0.45/0.42±0.11	17	0.37–0.47	0.43/0.43±0.03	20
Leg I basitarsus, depth	0.06–0.09	0.08/0.08±0.02	19	0.06–0.10	0.09/0.08±0.01	20
Leg I basitarsus, length/depth ratio	4.89–7.50	5.13/5.11±1.42	17	4.44–7.83	4.89/5.15±0.78	20
Leg I telotarsus, length	0.48–0.63	0.55/0.53±0.13	18	0.40–0.62	0.56/0.55±0.05	18
Leg I telotarsus, depth	0.07–0.10	0.08/0.08±0.02	18	0.07–0.10	0.08/0.08±0.01	16
Leg I telotarsus, length/depth ratio	6.11–8.43	6.87/6.49±1.73	17	5.00–8.86	6.75/6.66±1.04	16
Leg IV trochanter, length	0.50–0.63	0.55/0.52±0.13	17	0.49–0.65	0.55/0.56±0.04	19
Leg IV trochanter, depth	0.17–0.28	0.22/0.21±0.06	17	0.19–0.28	0.22/0.23±0.03	19
Leg IV trochanter, length/depth ratio	1.79–3.11	2.59/2.41±0.71	16	1.96–2.95	2.50/2.48±0.31	19
Leg IV femoropatella, length	1.44–1.75	1.62/1.51±0.40	16	1.40–1.75	1.58/1.56±0.08	17
Leg IV femoropatella, depth	0.18–0.30	0.23/0.22±0.06	17	0.21–0.31	0.24/0.24±0.03	16
Leg IV femoropatella, length/depth ratio	5.47–8.72	6.91/6.45±1.83	16	5.10–7.33	6.55/6.47±0.67	15
Leg IV tibia, length	1.40–1.64	1.49/1.41±0.36	17	1.32–1.56	1.43/1.44±0.08	17
Leg IV tibia, depth	0.12–0.17	0.13/0.13±0.03	19	0.11–0.16	0.13/0.13±0.01	18
Leg IV tibia, length/depth ratio	9.33–12.67	10.97/10.45±2.76	17	9.64–13.00	11.50/11.35±0.85	17
Leg IV basitarsus, length	0.51–0.64	0.56/0.54±0.14	17	0.49–0.60	0.56/0.55±0.03	18
Leg IV basitarsus, depth	0.10–0.13	0.11/0.10±0.03	19	0.09–0.13	0.11/0.11±0.01	18
Leg IV basitarsus, length/depth ratio	4.25–5.82	5.27/4.95±1.33	17	4.46–6.44	5.05/5.19±0.51	18
Leg IV telotarsus, length	0.81–0.93	0.87/0.82±0.21	16	0.74–0.96	0.85/0.85±0.06	17
Leg IV telotarsus, depth	0.09–0.11	0.10/0.10±0.03	17	0.09–0.13	0.11/0.10±0.01	18
Leg IV telotarsus, length/depth ratio	7.73–10.33	8.24/7.94±2.24	15	6.25–10.67	8.27/8.28±1.24	17

**Adults.** Body yellowish; carapace, chelicerae and pedipalps light brown. Vestitural setae of body and pedipalps long and pointed. Carapace rectangular, without granulation, longer than broad, epistome and eyes absent, a subocular seta usually present (sometimes missing on one side). Tergites and sternites undivided. Chelicera with 5 setae on hand, 1 seta on movable finger, spinneret well developed in female, weak in male, rallum of 8 blades, 2 distal blades dentate. Anterolateral process of coxa I long, broad and apically pointed, medial process prominent, rounded, with strong denticles. Pedipalps slender, chelal fingers with normal number of trichobothria (8 on fixed and 4 on movable finger), sensillum *p*_1_ slightly distal of *st*, *p*_2_ nearer to *st* than to *sb*, situated close to dental margin. Palpal trochanter without tubercles. Legs elongated, I–IV with 2 tarsal segments (basitarsus and telotarsus separated). Subterminal setae of telotarsi I and IV with short, smooth, ventral rami and small dorsal denticles in distal part. Telotarsus IV with 2 long tactile setae, telotarsus I without tactile seta. Claws with a small dorsal denticle about one-third from base.

**Males (20 specimens studied).** Chaetotaxy of carapace: total 16–21 setae, posterior margin mostly with 4 setae, exceptionally with 3 setae in 2 males and 5 setae in 1 male, anterior margin mostly with 4 setae, exceptionally with 3 setae in 2 males and 5 setae in 2 males. Chaetotaxy of tergites I–X: 4, 4, 4–6, 4–6, 4–7, 4–6, 5–7, 5–7, 6–7, 5–7. Chaetotaxy of sternites IV–X: 7–15, 8–12, 8–11, 8–11, 9–11, 8–10, 7–9. Anterior genital operculum with 9–19 setae, posterior genital operculum with 29–38 setae in total, comprised of 19–26 medial and 8–15 marginal setae. Chelicera: fixed finger with 12–22 and movable finger with 10–20 unequally long teeth. Pedipalp: fixed finger with 133–172 unequally long teeth and movable finger with 106–152 equally long teeth. See Table 2 for measurements.

**Females (20 specimens studied).** Chaetotaxy of carapace: total 16–20 setae, posterior margin mostly with 4 setae, exceptionally with 2 setae in 1 female, anterior margin mostly with 4 setae exceptionally with 5 setae in 2 females. Chaetotaxy of tergites I–X: 4–5, 4–5, 4–6, 4–6, 4–6, 5–6, 5–7, 5–8, 5–7, 5–7. Chaetotaxy of sternites IV–X: 6–13, 6–10, 8–11, 8–11, 8–12, 8–11, 7–9. Anterior genital operculum with 7–13 setae, posterior operculum with 12–18 setae. Chelicera: fixed finger with 14–20 and movable finger with 11–19 unequally long teeth. Pedipalp: fixed finger with 131–171 unequally long teeth and movable finger with 118–149 equally long teeth. See Table 2 for measurements.

**Tritonymph (1 specimen studied).** With same general characteristics as adults. Chaetotaxy of carapace: total 17 setae, posterior and anterior margin with 4 setae each. Chaetotaxy of tergites I–X: 4, 5, 5, 4, 6, 6, 6, 6, 6, 6. Chaetotaxy of sternites IV–X: 8, 11, 10, 11, 10, 8, 8. Chelicera: rallum of 8 blades, 2 distal blades dentate, fixed finger with 14 and movable finger with 13 unequally long teeth. Pedipalps: chela with 7 trichobothria on fixed finger and 3 on movable finger; fixed finger with 112 equally long marginal teeth, movable finger with 97 equally long marginal teeth.

**Measurements of tritonymph.** Body length 3.43. Chelicera: 0.54/0.27 (×2.00); movable finger length 0.35. Pedipalps: trochanter 0.53/0.23 (×2.30), femur 1.10/0.19 (×5.79), patella 0.96/0.20 (×4.80), palpal hand 0.81/0.24 (×3.38), hand length without pedicel 0.73, movable finger length 1.26, movable finger and hand length ratio 1.56, palpal chela 1.95/0.24 (×8.13). Leg I: femur 0.59/0.10 (×5.90), patella 0.39/0.10 (×3.90), tibia 0.47/0.07 (×6.71), basitarsus 0.27/0.08 (×3.38), telotarsus 0.41/0.10 (×4.10). Leg IV: trochanter 0.38/0.18 (×2.11), femoropatella 0.96/0.18 (×5.33), tibia 0.88/0.11 (×8.00), basitarsus 0.35/0.10 (×3.50), telotarsus 0.57/0.12 (×4.75).

#### Note.

A description of this tritonymph was previously published by Ducháč (2004b). Because of damage to the specimen, it was not possible to measure the carapace and the trochanter of leg I.

**Deutonymph (1 specimen studied).** With same general characteristics as adults. Chaetotaxy of carapace: total 19 setae, posterior margin with 5, anterior margin with 4 setae. Chaetotaxy of tergites I–X: 3, 4, 5, 6, 6, 6, 6, 6, 6, 5. Chaetotaxy of sternites IV–X: 8, 8, 8, 8, 8, 8, 7. Chelicera: rallum of 6 blades, 2 distal blades dentate; fixed finger with 14 and movable finger with 11 unequal teeth. Pedipalps: chelal with 6 trichobothria on fixed finger and 2 on movable finger; fixed finger with 73 equally long teeth, movable finger with 66 equally long teeth.

**Measurements of deutonymph.** Body length 2.57. Carapace: 0.68/0.66 (×1.03). Chelicera: 0.41/0.24 (×1.71); movable finger length 0.26. Pedipalps: trochanter 0.38/0.19 (×2.00), femur 0.76/0.15 (×5.07), patella 0.62/0.20 (×3.10), palpal hand 0.57/0.27 (×2.11), hand length without pedicel 0.53, movable finger length 0.91, movable finger and hand length ratio 1.60, palpal chela 1.33/0.27 (×4.93). Leg I: trochanter 0.20/0.13 (×1.54), femur I 0.40/0.09 (×4.44), patella 0.26/0.08 (×3.25), tibia 0.29/0.07 (×4.14), basitarsus 0.16/0.07 (×2.29), telotarsus 0.27/0.08 (×3.38). Leg IV: trochanter 0.24/0.16 (×1.50), femoropatella 0.69/0.15 (×4.60), tibia 0.54/0.09 (×6.00), basitarsus 0.24/0.09 (×2.67), telotarsus 0.39/0.09 (×4.33).

#### Karyotype.

**Figure 5.**
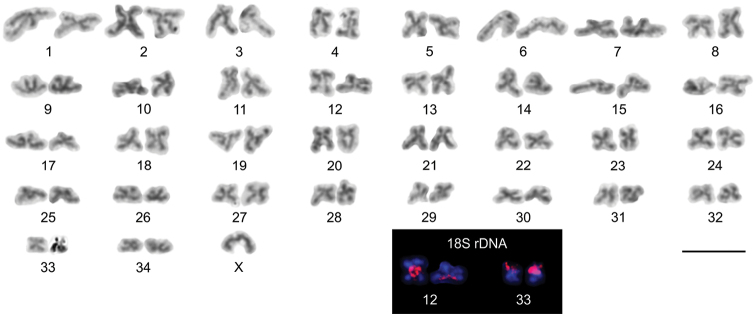
*Neobisiumslovacum*, male karyotype from Šingliarova chasm after Giemsa staining. Inset (black box) shows chromosomes detected by FISH with 18S rDNA (red signal). Based on two sister metaphase II plates. Scale bar: 10 µm.

The diploid complement of all three males analysed was 69 chromosomes (Fig. 5). The karyotype contains 19 pairs of metacentric (No. 2, 5, 8, 9, 13, 14, 16, 18, 19, 22, 23, 24, 26, 27, 29, 30, 32, 33, 34), ten pairs of submetacentric (No. 1, 4, 7, 10, 12, 17, 21, 25, 28, 31), three pairs of subtelocentric (No. 3, 11, 20) and two pairs of acrocentric (Nos. 6, 15) autosomes. The autosomes gradually decreased in length from 2.31% to 0.91% of the diploid set. An X0 sex chromosome system was identified in this species. The X chromosome shows an acrocentric morphology and constitutes 1.13% of the diploid set. Two pairs of 18S rDNA clusters were detected by FISH in this species. The 18S rDNA probe signals were localized close to the centromeres on the long arm of submetacentric chromosome pair No. 12 and on the short arm of metacentric chromosome pair No. 33 (Fig. 5).

## Discussion

The original description of *N.slovacum* by Gulička (1977a) included only a few morphological and morphometric features, but it was sufficient for recognition of the species. The recently examined specimens fit well with this description, except for minor variability in the chaetotaxy of the tergites and measurements of chelicera.

Ducháč (2004b) provided descriptions of the deutonymph and tritonymph, but the figures and table of measurements are missing on the web site of the journal and no printed version of the paper is available. According to the editor of the journal (Dr Eduard Stloukal, pers. com.), the figures and table were never published.

The redescription of previously studied and newly obtained material in the present paper gives a better assessment of intraspecific variability and adds some previously unstudied characters, such as the chaetotaxy of sternites, measurements of legs and the number of teeth on cheliceral and palpal fingers, presence of subocular setae, anterolateral and medial processes of coxa I, positions of chelal sensilla *p*_1_ and *p*_2_, form of subterminal seta, tactile seta of legs I and IV and denticulation of leg claws. The individuals show great variability in some characters, such as palpal femur and trochanter length, palpal chela length/width ratio, femur I and telotarsus I length/depth ratio, tibia IV and telotarsus IV length/depth ratio, femoropatella IV length/depth ratio in males, number of teeth on chelal and palpal fingers, chaetotaxy of sternites, and chaetotaxy of the male genital operculum. However, no significant differences were observed in chaetotaxy or measurements between populations of different caves. The greatest variability was observed in the palpal femur length/width ratio, with a range of 5.69–8.81 mm. Novák (2014) separated *Blothrus* species of the Carpathian Mountains in couplet 1 of his identification key between those with a palpal femur ratio of up to 6 and those, including *N.slovacum*, with a ratio of 6.5 or more. According to our measurements, this couplet will not always be reliable for identifying *N.slovacum*.

Concerning the karyotype, no differences were observed between the three males analysed. It should be noted that the material used for cytogenetic analysis comes only from two localities (Fig. 2) near each other in the northern part of the Plešivecká Plateau (Slovak Karst). In this case, dispersion of individuals between caves and hence gene flow between populations cannot yet be excluded. The number of chromosomes in *N.slovacum* (2n = 69) is similar to that of Neobisium (Neobisium) carcinoides (Hermann, 1804) (2n = 67; Sokolow 1926) and falls within the known range (2n = 30–71) reported in a preliminary analysis of species of this genus (Šťáhlavský et al. 2012b). Other cytogenetic features of *N.slovacum* are consistent with previous findings for pseudoscorpions. The chromosomes gradually decrease in length and their morphology is variable, which is typical for karyotypes of pseudoscorpions with higher number of chromosomes (Šťáhlavský et al. 2009, 2012a). The sex chromosome system is X0, which is assumed to be the ancestral state for pseudoscorpions (Troiano 1990). However, the morphology of the X chromosome is usually metacentric in this order (Šťáhlavský et al. 2012a, 2013, Kotrbová et al. 2016), whereas an acrocentric X was identified in *N.slovacum*. This morphology of the X chromosome was previously documented in only one population of *Olpiumpallipes* (Lucas, 1846) (Olpiidae) within pseudoscorpions (Šťáhlavský et al. 2006). It was supposed to be an effect of pericentric inversion from Greece in the population concerned. This type of chromosomal rearrangement can also be presumed for *N.slovacum*, because all other neobisiids thus far karyotyped possess biarmed X chromosomes (Šťáhlavský et al. 2013). Interestingly, two pairs of 18S rDNA clusters were identified in *N.slovacum*, even though one pair is considered to be the ancestral state for arachnids (Forman et al. 2013). However, a multiplication of the 18S rDNA clusters seems to be frequent, at least in arachnids with limited dispersal ability (Svojanovská et al. 2016, Šťáhlavský et al. 2018a). In such groups the variability in the number and position of rDNA clusters suggests intensive chromosomal changes that may also be expected in *N.slovacum*, in view of its subterranean habitat. It should be noted that no information is available about the number and position of rDNA clusters in any other pseudoscorpions, and the presence of two pairs of rDNA clusters might be typical for this order.

Summarizing all known distributional records, *N.slovacum* is currently known from 15 localities in the Slovakia and one locality in Hungary (Table 1 and Fig. 2). Based on this distribution, it can be expected to occur in the karst areas between the localities and near the border of the two countries. There are no valid records of species of the subgenus Blothrus in countries lying north of Slovakia in Europe or elsewhere. Beier (1936) described Neobisium (Blothrus) vulpinum Beier, 1936 from Krkonoše, western Sudetenland (a mountain range now within parts of the Czech Republic and Poland). However, Beier (1963) later stated that the locality details of the types were erroneous and synonymized this name with N. (B.) minutum (Tömösváry, 1883). The size of the caves and chasms occupied by *N.slovacum* range from small (Fialová cave, length 21 m; Vlčia chasm, length 29 m) to large (Zvonivá chasm, length 494 m; Diviačia chasm, length 468 m), situated at elevations between 258 and 795 m a.s.l. Air temperature in the caves, measured at the time of collection (using a digital thermometer), ranged from 5.4 °C (Zvonivá chasm) to 9.7 °C (Stará brzotínska cave). Air relative humidity during the collection of specimens was 94–100%.

## Supplementary Material

XML Treatment for Neobisium (Blothrus) slovacum
